# Update on the Mechanisms of Pulmonary Inflammation and Oxidative Imbalance Induced by Exercise

**DOI:** 10.1155/2016/4868536

**Published:** 2016-01-05

**Authors:** O. F. Araneda, T. Carbonell, M. Tuesta

**Affiliations:** ^1^Laboratorio Integrativo de Biomecánica y Fisiología del Esfuerzo (LIBFE), Escuela de Kinesiología, Facultad de Medicina, Universidad de los Andes, Monseñor Álvaro del Portillo 12455, Las Condes, 7620001 Santiago, Chile; ^2^Facultad de Biología, Laboratorio de Fisiología e Inmunología, Universidad de Barcelona, Avenida Diagonal 643, 08028 Barcelona, Spain; ^3^UDA Ciencias de la Salud, Facultad de Medicina, Pontificia Universidad Católica de Chile, Avenida Vicuña Mackenna 4860, Macul, 7820436 Santiago, Chile

## Abstract

The mechanisms involved in the generation of oxidative damage and lung inflammation induced by physical exercise are described. Changes in lung function induced by exercise involve cooling of the airways, fluid evaporation of the epithelial surface, increased contact with polluting substances, and activation of the local and systemic inflammatory response. The present work includes evidence obtained from the different types of exercise in terms of duration and intensity, the effect of both acute performance and chronic performance, and the influence of special conditions such as cold weather, high altitude, and polluted environments. Levels of prooxidants, antioxidants, oxidative damage to biomolecules, and cellularity, as well as levels of soluble mediators of the inflammatory response and its effects on tissues, are described in samples of lung origin. These samples include tissue homogenates, induced sputum, bronchoalveolar lavage fluid, biopsies, and exhaled breath condensate obtained in experimental protocols conducted on animal and human models. Finally, the need to simultaneously explore the oxidative/inflammatory parameters to establish the interrelation between them is highlighted.

## 1. Introduction

When doing physical exercise, the usual levels of organic performance are exceeded. However, we are designed to execute the exercise, depending on its variety, duration, intensity, and the environmental conditions under which it is done. The physiological and pathological processes will be activated, which can lead to the generation of an oxidative imbalance and the establishment of an inflammatory process [1, 2]. The oxidative damage happens as an additional cost of using oxygen to obtain energy and can occur when there is an increase in the formation of prooxidants and/or when the antioxidant defense decreases, causing an alteration of tissue product functionality of the structural damage to all the cellular components that contain lipids, carbohydrates, proteins, and nucleic acids [3]. Another response mechanism to physical stress is inflammation, which is triggered as a reaction to the mechanical damage of structural components (connective tissue; muscle, tendon, and bone) and nonstructural components (erythrocytes, endothelium, and epithelia) of the body [4–8]. As a result, stress hormones are released, such as cortisol and catecholamines, which activates the immune system, causing a particular response profile based on the release of soluble mediators (cytokines) and arachidonic acid derivatives (prostaglandins and leukotrienes). The latter and the stress hormones will cause changes in the number and activation of leukocytes subpopulations to the point that intense exercise of long duration can induce immune suppression (increasing the susceptibility to infection) [9], in contrast to the exercise of moderate intensity, which boosts the immune response. Both the alteration of the redox system and the inflammatory reaction have multiple points of interaction that have been previously evidenced [10–12]. The study of inflammatory/oxidative damage at a pulmonary level has been a topic poorly addressed [13–15], particularly in healthy humans and even more so in athletes. Most of the information in this subject arises from pathophysiology of pulmonary diseases, such as asthma, cystic fibrosis, and chronic obstructive pulmonary disease [16–27]. The lung has the crucial role of gas exchange and experiences great modifications of its activity during the exercise. This mobilizes larger volumes of air and modifies the breathing pattern from nasal to oral, increasing contact with a greater amount of pollutants that may be present in the environment. Also, the lung receives a greater amount of blood flow to increase the exchange in places that are well ventilated, which causes changes in the functioning of the vascular parenchyma [28, 29]. However, the anatomo-functional characteristics of the lungs make it very difficult to obtain information of the redox/inflammatory state in the different sectors of this organ. This work brings together the scientific papers that have addressed the phenomenon of altered pulmonary redox/inflammation environment induced by acute or chronic exercise, in a hypoxic environment, cold or contaminated, in both animal and human models, by focusing on the protocols and mechanisms that explain the phenomenon, as well as their potential implication on those who exercise.

## 2. Effects of Exercise on the Respiratory System and Its Relationship with the Generation of Oxidative/Inflammation Damage

**Figure 1:**
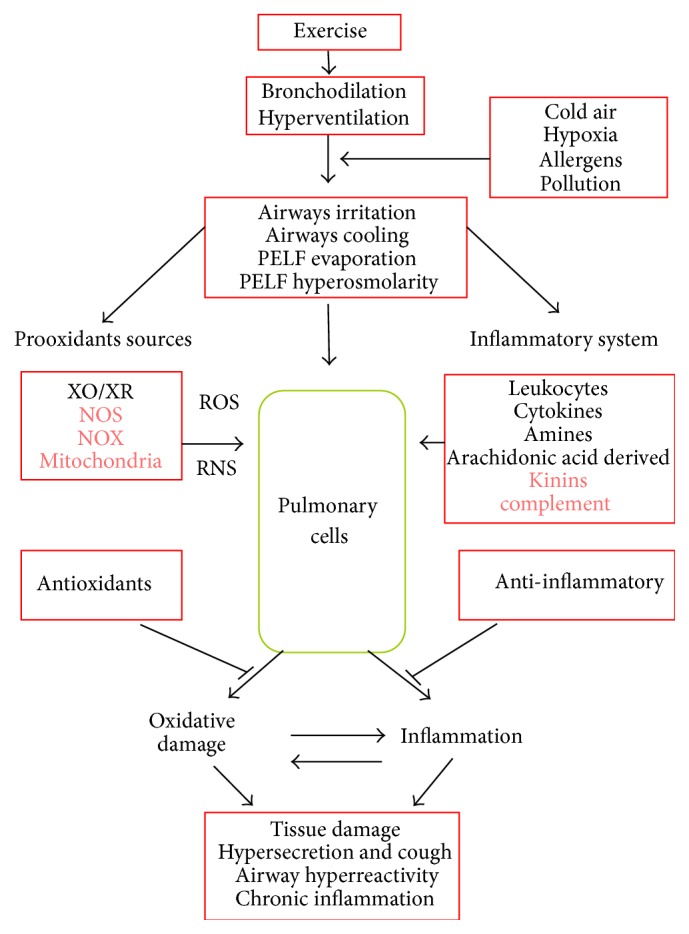
Proposed mechanisms related to the process of oxidative damage and pulmonary inflammation induced by exercise. Once the exercise starts the pulmonary ventilation increases and favors bronchodilation. This cools the airways, and also the part of PELF evaporates with subsequent increase of osmolarity and irritation appears. This activates the generating sources of free radicals and the inflammatory system. As a result of the foregoing, oxidative damage and a concomitant inflammatory process are potentially generated at pulmonary cell level; this may involve tissue damage, the increase of cough and the increased secretion of mucus, and the appearance of bronchoreactive phenomena and in the case that this stimulus is repeated (chronic exercise) to establish a process of chronic inflammation and remodeling of pulmonary tissue, particularly in the airways. This process is exacerbated when the exercise is performed in the presence of environmental conditions such as cold and hypoxia, in environments where pollen is abundant or in presence of contaminants (water/air). In red color the prooxidants sources and the parts of the inflammatory system that have not been studied are both appreciated. XO/XR = xanthine-oxidase/dehydrogenase; NOS = nitric oxide synthase; NOX = NADPH oxidase; PELF = pulmonary epithelial lining fluid.

When exercising, the mobilized air flow or pulmonary ventilation increases. This is explained by the increase of the respiratory rate, the tidal volume, and the appearance of bronchodilation. In addition to this, the pulmonary vascular bed will vasodilate to receive a greater blood flow. These changes, taken together, aim to increase gas exchange. Large air flows entering the lung during exercise will cause a modification of the breathing pattern towards one predominantly oral, favoring the evaporation of the fluid covering the pulmonary epithelium and the decrease of temperature of the airways. As a result, the pulmonary passages will cool down and the osmolarity of the epithelium will increase [30]. It should be noted that the cooling of the pulmonary passages as a result of the hyperventilation has been observed at comfortable environment temperature (+20°C) [31]. In this way, McFadden Jr. and Pichurko [31] showed a decrease of the tracheal temperature of 34°C at pulmonary ventilation of 15 L/min and of 31°C at 100 L/min. The cooling of the airway by hyperventilation produced by exercise is homologous to breathing cold air at rest. The latter is probably in the absence of air pollutants, the main irritative/proinflammatory factor of this region of our body. In cold environments, there is a greater amount of reports of respiratory symptoms [32] and chronic changes of epithelium similar to those of patients with chronically inflamed airways (e.g., asthmatics). Some authors observed, in humans, that the product of intense exercise appears to have similar symptoms to those observed in infection of upper airways [33–35]. However, with moderate training these symptoms decreased [36, 37]. It is probable that intense exercise of long duration, such as a marathon, will increase the susceptibility to infection of the airway by depression of the immune function, contrary to the effect caused by exercise of moderate intensity. Another factor involved in the oxidative/proinflammatory process of the airway is the greater contact with toxic particles and microorganisms present in the environment due to hyperventilation by exercise [38–40]. For example, the damaging effect on lung tissue of environmental substances such as chlorine, ozone, nitrogen oxides, particulate matter, and pollen is recognized [14, 41–43]. The entry of these substances by the pulmonary route can potentially generate systemic inflammation [44, 45] and this will affect the lungs. Finally, another factor of the recognized destabilizing effect of the oxidative balance and in favor of pulmonary inflammation is hypoxia [46, 47]. The general framework for the development of functional changes of the lung by exercise, the activation of the redox imbalance, and the inflammatory system are described in Figure 1.

## 3. Changes in Pulmonary Redox State and Exercise-Induced Inflammation

As mentioned previously, physical exercise induces changes in the redox/inflammatory state of the organism, at both systemic level and the different organs. In this regard, lung is one of the less studied organs in this context. In the following paragraphs, the most relevant results regarding pulmonary oxidative damage and inflammation caused by exercise are summarized. In this review, the work carried out in healthy subjects was privileged. Regarding the special conditions, hypoxia, water contaminants (chlorine), and cold have been included, leaving aside air pollutants, because there are several reviews regarding this subject [48, 49]. The details of the studies included in terms of goals, characteristics of the sample, the protocol used, and the results related to the pulmonary oxidative/inflammation damage by exercise are summarized in Tables 1 and 2 for human and animals, respectively.

## 4. Pulmonary Redox Balance and Acute Exercise

A direct relationship has also been reported during exercise, between the acute exercise intensity and the volume of exhaled nitric oxide (VNO), namely, volume minute (VE) multiplied by exhaled nitric oxide (eNO), for sedentary healthy [50, 60, 68, 69, 71, 82, 85–87, 90] and trained subjects [75, 89]. During exercise, eNO have been reported to be decreased when increasing VO_2_ [59, 75] and VE [75] in sedentary and active subjects [51, 60, 68, 69, 75, 82, 85, 86, 92]. In athletes, unlike Maroun et al. [75], Kippelen et al. [68] showed changes in eNO during exercise. In animal model, while exercising healthy horses, Mills et al. [112] observed a linear increase of the VNO as the oxygen consumption increased. After exercise, nitric oxide concentrations have shown controversial results. In swimmers, Bonsignore et al. [57] reported a decreased eNO after 5 km (~179 min) in slightly chlorinated pool; when performing the same test at the sea no changes were observed in this pair but the same distance was maintained at the sea. In other studies, also a decreased eNO after exercise has been observed in healthy subjects [64, 70, 88, 91]. However, in youngsters not trained in swimming, Carbonnelle et al. [58] found increases of eNO after swimming 2 sessions of ~1300 m in 45 min in a pool sanitized with electrical process (nonchlorinated water). Also, De Gouw et al. [61] found an increased eNO in healthy subjects after cycling for 6 min using dry air, while ventilation was kept constant in 40–50% of his or her predicted maximal voluntary ventilation (35 × FEV_1_). Other studies showed no changes in the eNO after exercise; Font-Ribera et al. [65] found no differences in eNO concentrations in pool swimmers; the same occurred with eNO in swimmers after an exercise of 45 min [81] and in healthy subjects after either cycloergometer [66, 94] or treadmill incremental exercise test [80].

Through the exhaled breath condensate (EBC) analysis, to observe the oxidative effects of the moderate acute exercise, Nowak et al. [79] subjected a group of healthy subjects to a submaximal exercise on cycloergometer during ~6 min; they found no changes in H_2_O_2_ and thiobarbituric acid reactive substances (TBARs). Araneda et al. [46] found no changes of H_2_O_2_ in EBC after three maximal cycle ergometries of 1 min in elite cyclists carried out at 670 and 2160 masl, but malondialdehyde (MDA) was higher at 2160 meters. Marek et al. [72], in two submaximal cycle ergometries to 60 W (~7 min) and 120 W (~5 min), and later in maximal exercise (~13 min), found no differences in H_2_O_2_ concentration in EBC [73]; however, in both studies, increases were found in the flow of formed H_2_O_2_ after exercise. On the same prooxidant, Mercken et al. [76] found an increase after maximal cycle ergometry in healthy subjects, with increments of 10 w/min, but they did not find any differences in subjects with chronic obstructive pulmonary disease after exercise. However, in another study they found no differences in H_2_O_2_ when healthy subjects performed a cycle ergometry with one leg (40%  *P*_max_) during 20 min [77]. Marek et al. [74] found that, after 50 min of high intensity running developed at ~18°C and $\begin{array}{c} \sim -15^{{}^{\circ}}C \end{array}$ of environmental temperature, the concentration and production rates of H_2_O_2_ in EBC were higher when the exercise was carried out in a cold environment. Recently an increase in H_2_O_2_ and nitrite concentrations and correlations between both metabolites in the EBC of 21 and 42.2 km race participants were found. Also in this study, while nitrite increased in EBC, plasmatic nitrite showed no modifications and no correlations between these variables, which suggests a probable localized origin of this process [53].

Until now, only two studies have determined one of the potential sources of prooxidants; thus, it has been described as an increment of xanthine oxidase activity in the pulmonary homogenate of rats that performed strenuous exercise (~15 min) on a treadmill (20 m/min), besides MDA and NO [111]. Likewise, Huang et al. [110] observed an increase of the activity of xanthine oxidase and lung MDA in older rats after running on a treadmill until fatigue, during ~63 min at 70%  VO_2_max. Prigol et al. [115] and Akil et al. [105] found increases in TBARs in rats that swam for 20 min and 30 min, respectively, while Reddy et al. [114] found increases in MDA in rats with a vitamin E deficient diet that swam until fatigued. Also in rats, increases of TBARs after swimming during ~2.5 h until fatigue were found [106]. The same result was found in pulmonary homogenates of untrained rats which swam until exhaustion [119]. A strenuous exercise protocol of ~66 min (80–85%  VO_2_max) showed no changes in TBARs in rats [107].

In healthy horses, no differences were observed in isoprostane 8-epi-PGF2*α* of supernatant of bronchoalveolar lavage fluid (BALF) after 50 min of running [13]. An increment of carbonyls in the lungs of rats was observed by Radák et al. [113] after an exercise till exhaustion on the treadmill. However, after an hour of a moderate intensity run in young and old rats, no changes were observed in the lung carbonyls [109].

With regard to the pulmonary antioxidant enzymes, after an hour of acute moderate exercise protocols on treadmills, young rats' lungs showed an increase in the activity of enzymes superoxide dismutase (SOD) of the type CuZn-SOD, Mn-SOD, of the catalase (CAT), without changes in the glutathione peroxidase (GSH-Px). The mRNA expression for these enzymes did not show differences [109]. Lin et al. [111] found an increase in SOD and glutathione reductase (GR) activity with no changes in CAT and GSH-Px activity in rats that ran at 30 m/min and 10% slope until fatigued. Finally, acute and prolonged exercise (more than an hour) at 80–85%  VO_2_max showed no changes in the activity of GSH-Px and SOD [107]. In acute exercise protocols, using swimming, Reddy et al. [114] found an increase in SOD and glutathione transferase (GST), while mild decreases in GSH-Px activity were observed in rats that swam until fatigued. Prigol et al. [115] found increase in CAT activity in rats that swam for 20 min. In rats that exercise for an hour, Terblanche [116] found increased CAT activity without differences between males and females. In rats 18 months old, Huang et al. [110] described an increase of SOD activity and the maintenance of levels of CAT, GSH-Px, and GR after 51 min on treadmill at 70% of  VO_2_max. Strenuous exercise increased the activity of GSH-Px, with no changes in GR [119]. In a report of Al-Hashem et al. [106], rats that exercised until fatigue decreased the activity of SOD and CAT.

Acute exercise has also altered the levels of nonenzymatic antioxidants; an increase of uric acid has been described, with no changes in total glutathione, in GSH, and in GSSG in BALF, after 50 min of incremental exercise in healthy horses [13]. In a study of rats that ran during ~81 min at 70–75%  VO_2_max until fatigue, no variations were found in the homogenized lung GSH [111]. In rats that swam until fatigue (~2.5 h), no differences were found at 600 m of altitude, but there was a decrease of GSH levels at 2270 meters [106]; in this same report, it was found that supplementation with nonenzymatic antioxidants such as VitC (20 mg/kg) and VitE (20 mg/kg), a single dose one hour before starting the exercise, decreases pulmonary lipid peroxidation and SOD and CAT activities increases, in both altitudes. Additionally, supplementation shows higher levels of GSH compared to animals not treated in altitude [106].

Thus, the increase in lung prooxidants and its consequences (lipid peroxidation) due to acute exercise appear to be related to the high intensity and duration of the effort, in terms of either minute ventilation or oxygen consumption, and are enhanced by a hostile environment (hypoxia, pollution, cold, etc.). However, a mainly enzymatic antioxidant adaptive response is still controversial. In contrast, the use of vitamin reducers (C and E) allows the antioxidant capacity to be increased and oxidative damage to be controlled (see Tables 1(a) and 1(b)).

## 5. Pulmonary Redox Balance and Chronic Exercise

In a first study of pulmonary prooxidants and chronic exercise, Carraro et al. [97] found no differences in eNO of child swimmers (trained 1 h/week during 6 months). Martin et al. [102] observed no differences in eNO of athletes based in pool and not based in pool exposed to pool environment during 5 and 0.5 h/week, respectively. For oxidative damage, Heinicke et al. [47] found a tendency towards increase of 8-isoprostanes in the EBC of biathletes who trained at 2800 meters during 6 weeks (4–6 h/d with 1 d/weeks of rest), which included extensive cross-country skiing, strength training, and shooting technique training.

In a model of physical training of rats, which jogged in 3 months a total of 24 sessions of 20 min/d at 60%  VO_2_max, no differences were found in pulmonary carbonyls, nitrite, or TBARs [121]. After 24 weeks of training at 50%  V_max_ for 60 min/d for 5 d/week, ROS decreased in BALF and no changes of increase were found in pulmonary 8-isoprostanes in trained mice [126]. Using the same load and frequency as before, the levels of eNO and MDA were not altered in lung homogenates of rats trained during 5 weeks [15]. However, during the 8 weeks of training in rats that swam with a 2% of additional body weight during ~50–80 min, an increment of pulmonary carbonyls and MDA was observed [119]. Gündüz et al. [122] found increases of TBARs in older rats (21 months) versus young rats (9 months), without any variations between old rats which were either trained or untrained in swimming during 12 months 1 h/d for 5 d/week. Altan et al. [117] found increases in MDA in rats trained at 3000 meters of altitude (120 min/d for 4 d/week during 9 weeks) compared to sedentary control rats and the ones not trained maintained at sea or height level. In Sprague-Dawley rat that was trained during 8 weeks on a treadmill, an increase in pulmonary TBARs and protein carbonyls was observed [123]. Regarding oxidative stress on nucleic acids, Asami et al. [118] found increases in 8-hydroxydeoxyguanosine in rats after a forced race on treadmill for five weeks in daily sessions with a gradual increase in the time of 30–90 min.

The chronic exercising has also had as a subject of study the potential changes of the expression/activity of the enzymes and nonenzymes pulmonary antioxidant. Likewise, Reis Gonçalves et al. [15] found an increase in the lung Mn-SOD expression of mice subjected to five weeks of training at moderate intensity (60 min/d in 3 d/wk); however, no changes were observed in the GSH-Px, GR, GST, and CAT activities. In another study, Olivo et al. [125] observed an increased expression in pulmonary CuZn-SOD and Mn-SOD postmaximal exercise test of trained mice during 4 weeks at 50% of the maximal speed on treadmill. Altan et al. [117] found increases of SOD activity after nine weeks of progressive training in a normobaric environment (5 to 30 min/d for 4 d/week), with no differences with a trained group at 3000 meters of altitude. da Cunha et al. [121] observed a higher pulmonary CAT activity in the ones trained on a treadmill during 12 weeks at 60%  VO_2_max (20 min/d), compared to control rats. In another study, Menegali et al. [124] found an increase of the CAT and SOD activity in lung of trained rat in swimming during 8 weeks. In mice trained on a treadmill for 24 weeks at 50%  V_max_ (60 min/d and 5 d/week) increases of GSH-Px were observed without changes of expression of CuZn-SOD, Mn-SOD, and Ec-SOD, studied in sections of pulmonary tissue [126]. In another study, older animals of 21 months that were trained for a year (1 h/d and 5 d/week) had a greater amount of SOD in comparison to control rats of their same age and to young rats. No differences were found in CAT activities, while GSH-Px had a greater activity than a group of their same age [122]. Finally, Aydin et al. [119] observed a decrease in the concentrations of GSH and an increase of GSH-Px activity in pulmonary homogenates of rats, after eight weeks of swimming with overload and progressive weekly time increment (50–80 min).

This reflects the fact that oxidative stress induced by chronic pulmonary exercise in animals is closely associated with high-intensity protocols, but not with those of moderate intensity (see Table 1(b)). However, when moderate chronic exercise was executed while at high altitude, both human and animals presented pulmonary oxidative damage (see Tables 1(b) and 2(b)). In contrast, antioxidant adaptation seems to be more closely related to the animal training time, with an increase in the activity of SOD and CAT in the medium term and the expression of SOD in the short term (see Table 2(b)).

## 6. Acute Exercise-Induced Lung Inflammation

In horses, Kirschvink et al. [13] found no cellular count variation in BALF after 50 minutes of exercise. In runners' sputum of 10 km (~35.4 min), 12 km (~46.1 min), and 21 km (~89.1 min) a trend of increasing polymorphonuclear neutrophils (PMNs) in samples of induced sputum was found [40]. In the same direction, Bonsignore et al. [56] reported a higher percentage of PMNs in induced sputum, compared to values previous to exercise and an increase in these cells after the marathon (~179 min). Also in induced sputum of runners, Denguezli-Bouzgarrou et al. observed in 2006 [62] and 2007 [63] an increase of PMNs after 60 minutes of moderate racing. In the latter study, higher concentrations of histamine, interleukin-8 (IL-8), LTB_4_, and LTE4 were also detected, subsequent to acute exercise during the precompetitive phase versus the competitive phase [63]. Chimenti et al. [5], in a 20-kilometer race (~90 min), reported an increase in IL-8 in the supernatant. Races in smaller time frames (~18 min) showed no changes in the amount of PMNs in induced sputum [93]. In rowers, after a short test of high intensity (1000 m in ~3 min), there was a trend towards an increase of epithelial cells and a positive association between the pulmonary ventilation/body weight (L/kg) and macrophages in induced sputum [78]. In swimmers, increases in lymphocytes and eosinophils and a decrease in macrophages were observed in induced sputum, after a 5 km race in the ocean (hypertonic environment) in relation to the same test performed in an open pool with low concentration of chlorine. However, there is no evidence of the increase in inflammatory cell activation [57]. In a chlorinated pool, in high performance swimmers, no changes were observed in the cellular composition of the induced sputum and the pH in EBC after 45 min at moderate intensity [81]. Larsson et al. [32] found an increase of granulocytes and macrophages in subjects that performed one hour of exercise, on a treadmill, at −23°C, without IL-8 changes in BALF samples. Derivatives of arachidonic acid have been studied in three works; thus, in a maximum acute exercise of approximately 12 min, increases in E_2_ prostaglandin and B_2_ thromboxane in EBC after exercise were found in men [83]. The leukotrienes in EBC were studied by Bikov et al. [54]; thus, after an eight-minute test on a treadmill no differences in the concentration of cysteinyl leukotrienes were found in normal people. In a test of 4 km of cycling with a 12% hill sloping during ~7 min, an increase of leukotriene B4 in BALF of athletes was found in comparison to the control subjects [67]. Also in EBC, Zietkowski et al. [95] found no changes in high sensitive C-reactive protein after 9 minutes of cycle-ergometry at 85% of  HR_max_ in healthy subjects.

The pH in EBC (EBC_pH_) is a potential marker of pulmonary inflammation that has been used in pathologies that have this condition. In acute exercise, the results have been variable; thus, Marek et al. [73] did not find differences after an exercise until fatigue (~13 min) in  EBC_pH_ of amateur athletes. Bikov et al. [55] did not observe changes in the  EBC_pH_ of healthy subjects after exercise, while there are other reports that show increases in pH after outdoor exercise [128] and after low-intensity (60%  HR_max_) exercise (~30 min) in nonathlete healthy subjects [84]. In races up to 10 km, no changes have been reported up to 80 min after the race, in both amateur runners [52] and physically active runners [53]. However, there are inverse correlations between changes in prooxidants and changes of  EBC_pH_ [53]. In distances that exceed 21 and 42 km, ~101 min and ~246 min, respectively, an acute decreasing trend of  EBC_pH_ was observed [52]. However, in an animal study conducted in horses, the group of Cathcart et al. [108] found an increase in  EBC_pH_ after running 1.6 km.

In summary, the majority of published papers demonstrate the infiltration of inflammatory cells (macrophages or granulocytes) after acute exercise in humans. A factor that probably influences this is the duration of the exercise, as the increase in PMNs was found only in protocols involving longer periods (see Table 1(a)). Cellular infiltration was found to be due to cold or chlorine. The role of exercise training is difficult to assess, given that the studies were conducted almost exclusively in trained subjects. We must add to this the reported changes in soluble inflammatory mediators. As a whole, these could be an expression of an asymptomatic acute inflammatory process similar to that observed in other tissues (muscle tissue). This would happen in a self-limiting way whenever the necessary conditions of time, environmental factors, and intensity are encountered.

## 7. Chronic Exercise-Induced Lung Inflammation

Studies in animals have shown that training during 120 min/d for a week on treadmill at 25 m/min increases the expression of mRNA to tumor necrosis factor-alpha (TNF-*α*) together with promoting a decrease of interferon gamma in pulmonary tissue samples [127]. Chimenti et al. [120] trained mice at moderate intensity for 6 weeks (5 d/week), showing leukocyte infiltration in the airway. At this level of epithelia, an increase of apoptosis and a decrease of the ciliated cells were also observed. In mice that trained 60 min/d to 50%  V_max_ for 24 weeks (5 d/week), no variation was observed in the number of macrophages in BALF, but it was possible to see a decrease of the capacity of these cells to form free radicals [126]. However, it is possible that the elaboration of training programs at moderate intensity (66%  VO_2_max) generates a reduction of the inflammatory response after the completion of ischemia and pulmonary reperfusion, which was evidenced as a decrease of the release of interleukin 1*β* and tumor necrosis factor-alpha (TNF-*α*) at plasmatic level in a model performed in rats [129]. An analogous result was described by Toledo et al. [126], who did not find differences in TNF-*α*, interleukin 10, monocyte chemotactic protein, and interleukin 1 receptor antagonist, quantified in lung sections of mice, after training to 50%  V_max_ for 1 h/day, 5 days per week, for 24 weeks.

In studies conducted in humans, it has been reported that the participation in a long distance race training program over the course of a year generates a persistent inflammatory process with no apparent clinical repercussion and an increase in PMNs and in IL-8 concentrations, leukotriene E_4_, and histamine in the supernatant of induced sputum samples [130]. Subjects who participated in high performance athletic training in sessions of 1 h/day for 10 days, interspersed with rest 5 days, had lower pH values in EBC compared to healthy control subjects [98]. The same result in this parameter was reported in runners by Greenwald et al. [128]. In the same direction, in amateur runners (~50 km/week) low levels of pH were reported compared to values of healthy control subjects [52]. High performance pool swimmers showed no differences in basal inflammatory parameters when compared with non-pool-based athletes; however, the analysis of the subgroup of athletes that had a positive result in the voluntary hyperventilation test (exercise-induced bronchial* hyperreactivity* indicator) presented a higher concentration of eNO and a higher count of eosinophils and of epithelial cells when compared to the group that had negative results on this test [102]; among other factors, this could be related to the number of years of practice of pool swimming, since no differences in eNO, in EBC pH, and in cellularity of induced sputum in adolescents were found when compared to normal subjects [131]. Elite swimmers, who trained between 800 and 3380 km/year, had more eosinophils and PMNs in induced sputum compared to nonathlete control subjects [99]. The cessation of the training for 3 months of swimmers decreases eosinophils and lymphocytes in induced sputum compared to active swimmers (~1870 km/year) [100]. The comparison between healthy athletes who are swimmers and others who are engaged in land exercise has shown an increased number of PMNs in induced sputum samples [96]; the same comparison showed no differences in PMNs and eosinophils in induced sputum [102]. Chronic inflammation can be associated with pulmonary epithelial damage; thus, increases of clear cell protein (CC16) in plasma of swimmers who trained during 20 weeks in a chlorinated pool have been reported [132].

In skiers, who trained 435 h/year, increase of lymphocytes and mast cells has been found, with no differences in the concentration of TNF-*α* and myeloperoxidase in BALF compared to nonathlete control subjects [103]. Karjalainen et al. [101] reported, through the study of bronchial biopsies, an increase in neutrophils, eosinophils, macrophages, and T lymphocytes in elite skiers (435 h/year) compared to healthy control subjects, along with air tract remodeling indicators as an increase in collagen I and collagen III deposits in the submucosa, a hyperplasia of racket cells, and a higher expression of type 5 mucin. The use of anti-inflammatories (800 micrograms/day of budesonide) by cross-country elite skiers (~427 h/year) during 20 weeks did not generate differences regarding the placebo (~468 h/year) in the cellularity (PMNs, macrophages, lymphocytes, eosinophils, and mast cells), studied in BALF and in endobronchial biopsy [104].

In summary, animal models of physical training show increases of soluble inflammatory mediators, which include TNF-*α*. Human studies have focused on subjects who have greater contact with irritants in the airway due to the specificity of their sport, whether runners (large ventilation volumes), skiers (cold), or swimmers (chlorine gas in the pool room). In these subjects, permanent tissue infiltration of granulocytes, macrophages, and lymphocytes has been observed. Evidence of these changes has been found in both noninvasive samples, such as induced sputum, and in biopsies in the bronchial region. At the same time, an increased presence of soluble proinflammatory substances has been reported. Overall, this suggests that these athletes in particular may suffer from persistent changes in tissue (chronic inflammation and airway remodeling) that have been associated with pulmonary symptoms and functional changes (see the bottom of Figure 1).

## 8. Oxidative Damage and Inflammation, Relations, and Potential Effects

The generation of prooxidant substances and the establishment of tissue oxidative damage are closely associated with inflammatory processes; thus, inflammatory cells are a known source of prooxidants derived from both oxygen and nitrogen [133]. At the same time, the increase of prooxidants has been involved in the intracellular signaling which leads to inflammatory cell activation, increased secretion of soluble mediators of inflammation [134], endothelial activation, and also increased expression of adhesion molecules and endothelial permeability [135]. This relation implies that, in many situations, the increase of prooxidants participates in the activation of inflammation and vice versa, demonstrating the close relationship between both phenomena [134]. The establishment of both oxidative damage and inflammation in the lungs has been involved in the origin/evolution of various pathological states; for example, both phenomena are a fundamental part of adult respiratory distress [136], asthma [137], chronic obstructive pulmonary disease [138], pulmonary hypertension [139], and viral infectious processes [140]. In the lungs, the relationship between oxidative changes and inflammation has rarely been studied as a main goal, but it is presumed that, in view of the studies conducted in other organs, it must be closely related. This is particularly important in subjects practicing sport, as both inflammation damage and oxidative damage have been implicated in the pathogenesis of phenomena of high prevalence in athletes such as rhinitis, bronchial hyperreactivity, asthma, and airway remodeling [27, 141]; so, most respiratory symptoms (coughing, wheezing, breathlessness, and chest tightness) in endurance athletes such as cross-country skiers are known [142]. In addition, cross-country skiers show a presence of PMNs and lymphocytes infiltration in the airways [101]. This phenomenon can also be extrapolated to other endurance athletes [143] such as marathon runners, cyclists, and swimmers, the latter of which are also exposed to the chlorine in swimming pools, which could be one of the main factors inducing increased eosinophils and leukocytes in the sputum.

## 9. Methods for the Study of Lung Inflammation/Oxidative Damage by Exercise

The study of the oxidative/inflammatory damage in the lungs is challenging due to both anatomic functional limitations and the limitations of currently applied techniques. Current evidence on this topic focuses primarily on the study of lung diseases, while studies on the effect of exercise as a trigger effect of this phenomenon in healthy people are scarce. Summarizing what is known to date for the species analyzed, the determinations made and the samples obtained are shown in Tables 1 and 2. Lung tissue microenvironment has challenged developers of study methodologies, so, although systemic markers have been proposed (CC16, surfactant proteins A and B, and Krebs von den Lungen-6), they do not yet have sufficient capacity to indicate minor damage, which implies that the processes of the lung itself cannot always be ascertained. For this reason, it is preferable to test samples originated from the lung; those currently under study are exhaled breath (whether direct or condensate), fluids (BALF, induced sputum, and nasal lavage), and cells and portions of whole tissue (biopsies, tissue homogenates, and cut pieces of tissue). Unfortunately, today there is still much controversy regarding the interpretation of the results obtained with these methods. In relation to oxidative/inflammatory exercise phenomenon, in animals, exhaled breath [112], lung tissue homogenates [113, 114, 117, 118, 120, 121, 127], bronchoalveolar lavage [121, 126], and lung tissue sections [126] have been used. In humans, most methods are focused on noninvasive methods and, among these, the induced sputum is the most widely used [40, 56, 57, 62, 63, 78, 81, 93, 96, 99, 100, 102, 144]. Another sample studied corresponds to exhaled breath, which was analyzed whether directly [56, 57, 59, 65, 71, 75, 81, 89, 97, 102] or after being condensed at low temperature [46, 53, 65, 72–74, 77, 79, 81, 83, 84, 128, 139]. Very few studies have used bronchoalveolar lavage [32, 103, 104] and lung tissue obtained by endobronchial biopsy [101, 104].

## 10. Discussion

In summary, we found that in acute exercise (see Tables 1(a) and 2(a)) there is more evidence of changes in cellularity (predominantly granulocytes) when it (was) is a prolonged high-intensity exercise. This change was not so evident in animals; however, this should be resolved in further studies because it is a parameter measured recently in this population. Long-term of acute moderate exercise (>60 min) in humans stimulated an increase of pulmonary inflammatory mediators (IL-8, LTB_4_, and LTE_4_). Now, regarding prooxidants, a systematic increase in humans is observed after more than thirty minutes of exercise. It is noteworthy that, in acute exercise in animals, reports of an increase in lung lipid peroxidation are the majority, while it has not been observed in humans, except for intense exercise at high altitudes. This may be partially explained by the techniques used: while tissue samples were analyzed in animals, EBC samples were analyzed in humans; in another aspect, the change with greater support in relation to the enzymatic activity corresponds to the maintenance or decreased levels of GSH-Px and to the increase in SOD.

With regard to chronic exercise (training) and its effects (see Tables 1(b) and 2(b)), the number of studies is still very small, but there is a tendency observed, seen in humans, towards changes in cellularity compatible with chronic inflammation of the airways, particularly in subjects exposed to cold and chlorine. In animals, changes in pulmonary cellularity (leukocyte infiltration) were observed in only one study [120]. For soluble inflammatory mediators, in animals the scientific evidence has shown an increase in the concentration of these substances (IL4, IL6, and mRNA TNF-*α*) subsequent to chronic exercise. The oxidative damage was observed in animals following moderate chronic exercise (>4 sem), specifically in older rats, and cold or altitude environment. In humans, only one study showed oxidative damage by altitude training [45, 47]. With regard to enzymatic antioxidants, a tendency towards higher levels in SOD and GSH-Px is observed in humans. As for nonenzymatic antioxidants, only one study showed a decrease in the concentration of pulmonary GSH in trained rats [119].

The problem requires further study to clarify numerous questions in order to have a more definitive overview; thus, several challenges for researchers in the field have arisen. Likewise, the activity of the sources of production of free radicals in the lung (mitochondria, xanthine oxidase, NADH oxidase, and NOS) should be studied and the knowledge of the status of antioxidant systems, particularly in humans, where there are no records available, should be improved. Regarding inflammatory parameters, the study of soluble mediators of inflammation should be extended; in addition, the effect of both substances with antioxidant and anti-inflammatory effect should be explored. Furthermore, it is necessary to generate research projects which explore the parameters of oxidative/inflammatory mechanisms simultaneously in order to establish the interrelation mechanisms between both processes. It is also necessary to characterize the effect of time and intensity of performed exercise, the role of environmental conditions, and the level of training of the subjects on oxidative damage/lung inflammation by exercise. Finally, to advance the resolution of this problem, it is urgent to improve the technical conditions to allow obtaining representative samples of lung environment in its different compartments, and it is also necessary for these methods to be noninvasive and contribute to monitoring the athletes.

## Figures and Tables

**(a):** 

Author, year	Aim	Sample's characteristics	Exercise protocols	Samples obtained	Oxidative or inflammatory main results
Adachi et al. 1997 [50]	eNO and VNO in patients with CHF during exercise^*∗*^	CHF patients and healthy control subjects (C)	Maximal incremental cycloergometer test in CHF patients (10 W/min) and C (25 W/min) until exhaustion	EB	DE: ↑ VNO during exercise peak in C

Agostoni and Bussotti 2003 [51]	Correlation between eNO and lung mechanics during exercise in CHF^*∗*^	CHF patients and healthy control subjects (C)	25-W constant workload exercise cycle-ergometry test	EB	DE: ↓ eNO during 3rd and 5th minutes of exercise in C

Araneda et al. 2005 [46]	Lung oxidative damage from exercise at a medium altitude^*∗*^	Highly trained mountain bikers	Three repetitions of cycle-ergometries of 1 min at maximum intensity in 670 and 2160 MASL with breaks of 1 min	EBC and serum	PE: ↑ [MDA] in EBC, with no changes in serum at 2160 MASL

Araneda et al. 2012 [52]	Duration of a long distance exercise on pulmonary oxidative damage	Amateur runners	Urban 10 km (~53 min), 21 km (~101 min), and 42.2 km races (~246 min)	EBC	PE: ↑ [H_2_O_2_] and ↑ [NO_2_^−^] in 21 km and 42.2 km races and no changes in [MDA]; there was a tendency to ↓ of pH

Araneda et al. 2014 [53]	Pulmonary oxidative damage in long distance exercise	Healthy active subjects	10 km race in outdoor athletic track (~50 min)	EBC	PE: ↑ [H_2_O_2_], ↑ [NO_2_^−^]_EBC_/[NO_2_^−^]_Plasma_ with no changes in the [MDA]; there was a tendency to ↑ of pH

Bikov et al. 2010 [54]	Changes in [Cys-LTs] caused by exercise in asthmatic patients	Nonsmoking asthmatic patients (A) and nonsmoking healthy control subjects (C)	Race on treadmill at a speed and slope maintaining 80–90% HR_max_ (220 − age), which was regulated in 2 min and then maintained during 6 min	EBC	PE: with no changes in [Cys-LTs] in C, but ↑ in A

Bikov et al. 2014 [55]	Changes in EBC_pH_ during EIB in asthmatic patients^*∗*^	Asthmatics, who reported breathlessness following exercise, and healthy control subjects (C)	Exercise challenge test on a treadmill (details were not described by authors)	EBC and EB	PE: no change of pH in EBC in C

Bonsignore et al. 2001 [56]	Endurance exercise on inflammatory cells in AWs and eNO	Amateur runners	Marathon race (~179 min)	IS and EB	PE: ↑ PMN in IS and ↑ eNO in EB

Bonsignore et al. 2003 [57]	Swimming on inflammatory cells and eNO in the AWs	Swimmers (S) and healthy control subjects (C)	Swimming of 5 km only in the swimmers group, an open pool series (~70 min) and other series in the sea (~54 min)	IS and EB	B: >PMN and <MØ in the IS of S versus C PE: ↑ eosinophils, ↑ lymphocytes, and ↓ MØ in the sea versus swimming pool; eNO was > in the sea in comparison to swimming pool

Carbonnelle et al. 2008 [58]	eNO after swimming sessions^*∗*^	Trained healthy young people, not trained with swimming	Swimming in 2 sessions of 45 min (~1300 m), in a disinfected pool with [NaClO] and another sanitized with electrical process	EB	PE: ↑ eNO only in sanitized pool

Chimenti et al. 2009 [40]	Inflammation of the AWs in urban races in different climatic seasons^*∗*^	Amateur runners	21 km race in autumn (~89.1 min), 12 km race in winter (~46.1 min), and 10 km race in summer (~35.4 min)	IS	B: ↑ PMNs with ↑ [TNF-*α*] and ↑ [IL-8] PE: PMNs tended to ↑

Chimenti et al. 2010 [5]	Damage and inflammation of the lung epithelium in a long distance exercise	Amateur runners and healthy control subjects	20 km outdoor races (~90 min)	IS and serum	PE: ↑ [IL-8] in IS and ↑ CC16 in serum

Chirpaz-Oddou et al. 1997 [59]	eNO and VNO during exercise	Healthy control and trained subjects	Incremental cycloergometry to exhaustion with 5 min of passive recovery in sedentary subjects (♂ ~30 min and ♀ ~20 min) and trained subjects (~14 min)	EB	DE: ↓ eNO progressive with ↑ exercise intensity from 65% VO_2_max and ↑ VNO with the ↑ of the intensity of exercise > 30 W in all subjects

Clini et al. 2000 [60]	To evaluate eNO during exercise in patients with stable COPD^*∗*^	COPD patients and healthy control subjects (C)	Maximal cycle-ergometry test (cadence: 60 cycles/min and load: 10 W/min) until exhaustion	EB	DE: ↓ eNO at peak exercise and ↑ VNO in C

De Gouw et al. 2001 [61]	Role of eNO in the airway response to exercise by using L-NMMA, L-arginine, or placebo as pretreatment to exercise challenge^*∗*^	Asthmatic patients and healthy control subjects (C)	Cycle-ergometry for 6 min using dry air, while ventilation was kept constant in 40–50% of his or her predicted maximal voluntary ventilation (35 × FEV_1_)	EB	PE: ↑ eNO 30 min after exercise in C

Denguezli-Bouzgarrou et al. 2006 [62]	Endurance exercise and inflammatory cells of the AWs	Long-distance runners	Races on treadmill at 80% of MAS (~60 min)	IS	PE: ↑ PMNs, ↓ MØ, and ↑ lymphocytes

Denguezli-Bouzgarrou et al. 2007 [63]	Inflammatory mediators, cellular composition in AWs, and acute exercise during a sports season	Long-distance runners	Race at 80% MAS during the basic, precompetitive, and competitive period of a sport season in 1 year (~60 min)	IS	PE: ↑ PMNs in the precompetitive and competitive period. ↑ MØ in the precompetitive period; also, ↑ [histamine], ↑ [IL-8], ↑ [LTB_4_], and ↑ [LTE_4_] in the competitive phase

Evjenth et al. 2013 [64]	To investigate the effect on FE_NO_ of a standardized exercise challenge test on a treadmill^*∗*^	Nonasthmatic children with and without allergic rhinoconjunctivitis (AR) symptoms	Run on treadmill (6 to 8 min); heart rate target during the last 4 min was 95% of predicted maximum heart rate (220 − age)	EB	PE: ↓ eNO in nonasthmatic children without allergic rhinoconjunctivitis

Font-Ribera et al. 2010 [65]	Inflammation and postexercise pulmonary oxidative stress^*∗*^	Healthy subjects	Swimming in a chlorinated indoor-swimming pool (40 min), whose average speed was 22.5 ± 9.7 m/min	EBC and EB	PE: no changes of eNO in EB; [RANTES], [IL-12p70], [IFN-*γ*], [IL-4], [IL-8], [IL-10], [IFN-*γ*-induced protein 10], [TNF], [VEGF], and [8-isoprostane] in the EBC were not modified

García-Río et al. 2006 [66]	FE_NO_ before and after exercise challenge in patients with asthma and its relationship with airway obstruction^*∗*^	Nonsmoking, steroid-naïve, atopic patients with mild persistent asthma and nonsmoking, nonatopic, healthy subjects (C)	Performing an exercise challenge on a cycloergometer, with monitored ventilation (exercise parameters were not presented)	EB	PE: with no changes in eNO of healthy subjects

Hopkins et al. 1997 [67]	Pulmonary capillary pressure and function of the alveolar-capillary barrier during intense exercise^*∗*^	Athletes with signs of hemoptysis by exercise and healthy control subjects	4 km cycling with 12% hill sloping during ~7 min	BALF	PE: >alveolar MØ, >[LTB_4_], and < lymphocytes in athletes versus control subjects

Kippelen et al. 2002 [68]	eNO level in endurance-trained athletes during and after intense exercise^*∗*^	Nine athletes with exercise-induced hypoxaemia (EIH), 12 athletes without EIH, and 10 untrained subjects	15 min intense cycling exercise at 90% VO_2_max	EB	DE: ↓ eNO and ↑ VNO (last 3 minutes) in all groups

Larsson et al. 1998 [32]	Cold air and inflammation in the AWs during rest and exercise^*∗*^	Healthy subjects	Race on treadmill at −23°C and +22°C, each with 4 stages with 15 min at moderate intensity and 15 min of recovery	BALF	PE: at −23°C ↑ granulocytes and ↑ MØ; no changes in [IL-8]

Lovell et al. 2000 [69]	eNO and incremental exercise test in chronic congestive cardiac failure^*∗*^	Chronic congestive cardiac failure patients and healthy control subjects (C)	Performing Bruce protocol modified by inclusion of an initial 3 min stage at 5% incline, later performing a constant workload test (6 min at 2.7 km h^−1^ and 5% incline)	EB	DE: ↓ eNO and ↑ VNO during Bruce test in C; ↑ VNO during constant workload test

Mantione et al. 2007 [70]	eNO breath levels just before engaging in their respective activity	Healthy control subjects	Going up and down the stairs on a 20-foot staircase for 2 min	EB	PE: ↓ eNO 1 minute after exercise

Matsumoto et al. 1994 [71]	eNO and VNO during exercise	Healthy subjects	Cycle-ergometry at 100 W and maximum intensity with 5 min of recovery (~13 min)	EB	DE: ↑ VNO at 100 W and at maximum pedaling intensity

Marek et al. 2008 [72]	[L-lactate] and [H_2_O_2_] during exercise^*∗*^	Healthy subjects	Cycle-ergometer steady-state exercise at 60 W (~7 min) and 120 W (~5 min)	EBC	DE: ↑ [L-lactate] and ↑ [H_2_O_2_] in 60 W and 120 W

Marek et al. 2009 [73]	Maximal exercise, H_2_O_2_ release rate, and acid-base status	Amateur athletes	Incremental cycloergometry to exhaustion (~13 min)	EBC	PE: ↑ [H_2_O_2_] with no changes in pH nor [HCO_3_^−^]

Marek et al. 2013 [74]	Exercising in cold weather and release of H_2_O_2_^*∗*^	Healthy subjects	Races on treadmill at 75–80% HR_max_ at ~18°C and ~−15°C (~50 min)	EBC	PE: ↑ [H_2_O_2_] and ↑ rate of H_2_O_2_ release in both temperatures

Maroun et al. 1995 [75]	Physical condition and release of eNO during exercise	Healthy sedentary subjects (S), active subjects (Ac), and athletes (A)	Cycle-ergometries in steady-state at 1 and 2 L/min of VO_2_ only performing an additional one at 4 L/min of VO_2_	EB	PE: ↓ eNO at >VO_2_ in S and Ac; ↑ lineal of VNO with ↑ VO_2_ in A

Mercken et al. 2005 [76]	Exercise-induced oxidative stress in COPD^*∗*^	COPD patients and healthy control subjects (C)	Incremental cycle-ergometry exercise test until exhaustion and submaximal constant work rate exercise test (60% maximal power output)	EBC	PE: ↑ [H_2_O_2_] in maximal but not in submaximal exercise in C

Mercken et al. 2009 [77]	Pulmonary oxidative stress by endurance exercise in COPD and healthy subjects^*∗*^	COPD patients and healthy control subjects	Cycle-ergometry on one leg at 40% of maximum power output (20 min)	EBC	PE: ↑ [H_2_O_2_] in COPD patients but not in healthy control subjects

Morici et al. 2004 [78]	VE during exercise and inflammation in the AWs	Young rowers	Maximal run of 1000 m on the rower ergometer (~3 min)	IS	DE: ↑ tendency in epithelial cells at a higher VE PE: ↑ MØ with both ↑ VE/kg and ↑ VT/kg

Nowak et al. 2001 [79]	Prooxidants and oxidative damage by moderate exercise	Healthy subjects	Cycle-ergometer exercise test at 120 W during 6 min or until a HR of 120 bpm is reached	EBC	PE: with no changes in [H_2_O_2_] and [TBARs]

Nadziakiewicz et al. 2006 [80]	Effects of the physical activity on eNO levels in healthy subjects and in CAD patients^*∗*^	CAD patients and healthy control subjects smokers and nonsmokers	Bruce protocol exercise test	EB	PE: without changes in eNO in healthy control subjects nonsmokers

Pedersen et al. 2009 [81]	Inflammation in the AWs after 1-exercise session	High performance swimmers	Swimming in indoor-swimming pool at moderate intensity (45 min) whose average heart rate was 162 bpm	EBC and IS, EB	PE: no changes in the cellular composition in IS, eNO in EB, nor pH in EBC of swimmers

Pogliaghi et al. 1997 [82]	VNO after modifying pulmonary blood flow with head-out water immersion or increased gravity at rest and during exercise^*∗*^	Nonsmokers and healthy subjects who underwent air with normal conditions, water immersion, or increased gravity (1 Gz or 2 Gz)	Incremental cycle-ergometry test, loading was increased progressively by 50 W every 3 min until voluntary exhaustion	EB	DE: ↓ eNO and ↑ VNO in all groups

Pucsok et al. 2007 [83]	Lung PGE_2_ and TXB_2_ and exercise	Judo competitors	Incremental run on treadmill until VO_2_max is reached (run time was not recorded)	EBC	PE: ↑ [PGE_2_] and ↑ [TXB_2_] in ♂, but not in ♀

Riediker and Danuser 2007 [84]	Low-intensity physical activity and pH	Healthy subjects	Walk on treadmill at 60% HR_max_ predicted with 1 min pause every 10 min (~30 min)	EBC	PE: ↑ pH

Riley et al. 1997 [85]	NO production in patients with abnormalities of the pulmonary circulation^*∗*^	PPH (primary pulmonary hypertension), PF (pulmonary fibrosis), and normal subjects group	Maximal (20 W/min in the normal subjects and 15 W/min in the PF patients and individual estimated exercise tolerance in PPH patients) and submaximal constant work rate cycle-ergometry exercise test (work rate VO_2_ midway between each patient's anaerobic threshold and VO_2_max)	EB	DE: ↓ eNO and ↑ VNO in normal subjects at peak exercise in maximal and constant work rate exercise test

Rolla et al. 2003 [86]	Relationship between eNO and exercise tolerance in patients with moderate MS^*∗*^	Patients with moderate MS and healthy control subjects (C)	Symptom-limited incremental exercise test with an upright cycle-ergometer (25 W every 3 min until exhaustion)	EB	DE: ↓ eNO and ↑ VNO in all groups at the end of exercise

Shin et al. 2003 [87]	Relationship between exercise and NO exchange	Nonsmoking healthy adults	High-intensity exercise treadmill test at 90% of the predicted maximum heart rate (220 − age in years) for 20 min	EB	PE: ↑ VNO

St Croix et al. 1999 [88]	Effect of exercise on endogenous NO formation by measuring eNO at a constant airflow rate	Healthy, nonasthmatic, and nonsmoking subjects	3 min of constant-load cycle-ergometry exercise test at three different exercise intensities corresponding to 30%, 60%, and 90% VO_2_max	EB	PE: ↓ eNO and ↑ VNO for all intensities of exercise in healthy subjects

Therminarias et al. 1998 [89]	Exercise in cold air on eNO and VNO^*∗*^	Highly trained subjects (cross-country skiers, triathlon, and running)	Incremental cycloergometry to exhaustion in a climate chamber at +22°C and −10°C (~30 min)	EB	DE: ↓ eNO with the ↑ of the intensity >60 W in +22°C and ↑ VNO with the ↑ of the intensity >30 W in both temperatures

Trolin et al. 1994 [90]	eNO and VNO during exercise	Healthy subjects	Moderately heavy exercise on a cycloergometer (♀: 90 W for women and ♂: 150 W for ♂)	EB	DE: ↓ eNO

Tufvesson et al. 2013 [91]	Relationship between CC16 levels in plasma and urine after exercise with exhaled breath temperature and eNO^*∗*^	Asthmatic and healthy control subjects	During first six minutes speed and slope were adjusted to maintain the heart rate subject to 90% of their theoretical maximum heart rate (220 − age); the next two minutes were adjusted again to reach maximum effort	EB	PE: ↓ eNO in both groups

Verges et al. 2006 [92]	Effect of prolonged exercise on the NO concentration in the lung	Nonsmokers undertaking a moderate to intense training program participated in the study	100 min exercise test was performed on a cycle-ergometer (5 min of rest, 30 min warm-up at 25% *P*_max⁡_, 10 min at 60% *P*_max⁡_, 2 min at 25% *P*_max⁡_ repeated five times (S1 to S5), and 10 min of active recovery at 25% *P*_max⁡_)	EB	DE: ↓ eNO for all exercise sessions (WU, S1 to S5, and active recovery)

Wetter et al. 2002 [93]	EIAH and pulmonary inflammation^*∗*^	Endurance athletes with EIAH who used anti-inflammatory or placebo	Maximal incremental run on treadmill to exhaustion (~18 min)	IS	PE: with no PMNs, lymphocytes, nor MØ; ↑ [Histamine] in placebo

Yasuda et al. 1997 [94]	To examine the origin and role of eNO during exercise	Healthy control subjects	Two sets of 10 minutes in a cycle-ergometer (5 min without load and 5 minutes with 60 W and 60 RPM) separated, with 15 minutes between them	EB	DE: with no changes in eNO

Zietkowski et al. 2010 [95]	To assess the possible association of EIB with low-grade systemic inflammation in asthmatic patients^*∗*^	Asthmatics (14 with EIB, 10 without EIB) and healthy volunteers	Cycle-ergometer test for 9 min with a fixed workload adjusted to increase the heart rate to 85% of the maximum predicted for the age of each patient	EBC	PE: with no changes in hs-PCR in healthy volunteers

AWs: airways; BALF: bronchoalveolar lavage fluid; CAD: coronary artery disease; CC16: Clara cell secretory protein; CHF: chronic heart failure; COPD: chronic obstructive pulmonary disease; Cys-Lts: cysteinyl leukotrienes; EB: exhaled breath; EBC: exhaled breath condensate; EIAH: exercise-induced arterial hypoxemia; EIB: exercise-induced bronchoconstriction; eNO: exhaled nitric oxide; FE_NO_: fractional exhaled nitric oxide; HCO_3_^−^: bicarbonate; H_2_O_2_: hydrogen peroxide; HRmax: maximum heart rate; IFN-*γ*: interferon gamma; IFN-*γ*-induced protein-10: interferon-gamma-induced protein-10; IL-12p70, IL-4, IL-8, and IL-10: interleukin-12p70, interleukin-4, interleukin-8, and interleukin-10; IS: induced sputum; L-NMMA: N-monomethyl-L-arginine; L-lactate: lactate; LTB_4_: leukotriene B_4_; LTE_4_: leukotriene E_4_; MØ: macrophages; MAS: maximal aerobic speed; MS: mitral stenosis; MDA: malondialdehyde; MPO: myeloperoxidase; MASL: meters above sea level; NaCLO: sodium hypochlorite; NO_2_^−^: nitrite; NO output: nitric oxide output (eNO × VE); PGE_2_: prostaglandin E_2_; *P*_max_: maximal power output; RANTES: regulated upon activation, normal T-cell expressed, and secreted; TBARs: thiobarbituric acid reactive species; TNF(-*α*): tumor necrosis factor (alpha); TXB_2_: thromboxane B2; Se: selenium; VE: minute ventilation; VEGF: vascular endothelial growth factor; VNO: volume of nitric oxide; VO_2_max: oxygen uptake (maximal); VT: tidal volume. In “*Oxidative or inflammatory main results,*” DE: during exercise and PE: postexercise. In “*Aim,*” ^*∗*^the effect of exercise was not the primary aim of the study.

**(b):** 

Author, year	Aim	Sample's characteristics	Experimental protocols	Samples obtained	Oxidative or inflammatory main results
Belda et al. 2008 [96]	Type of sport (aquatic or terrestrial) and cell count^*∗*^	Elite healthy athletes and with asthma	Comparison of baseline samples between healthy and asthmatic athletes who practice water sports in pools or terrestrially (T: ~20 h/wk, with the exception of healthy subjects in water with T: ~10 h/wk)	IS	There was a positive correlation between PMNs with training time and water sport in the pool

Carraro et al. 2006 [97]	eNO in regular attendance to swimming pools^*∗*^	Children swimmers attending and control children not attending the swimming pool	Comparison of baseline samples between swimmers who attended a swimming pool (1 h/week/6 months) and control subjects	EB	There were no differences in eNO between both groups

Ferdinands et al. 2008 [98]	Exercise in contaminated environment and inflammation	Cross-country athletes and healthy control subjects	Comparison of baseline samples before and after 10 workouts in 15 d (~1 h/d)	EB	<pH in cross-country athletes compared to their control subjects between their respective sample times

Heinicke et al. 2009 [47]	Pulmonary oxidative damage and prolonged stay in medium height training^*∗*^	Biathletes and sedentary control subjects	Comparison of baseline samples between biathlete (T: ~5 h/wk) and control subjects; both groups were exposed to 2800 MASL during the 6 weeks	EBC	[H_2_O_2_] and [8-isoprostane PGF_2_ *α*] with no differences between groups; by gathering data ↑ [H_2_O_2_] and tendency to ↑ [8-isoprostane PGF_2_ *α*]

Helenius et al. 1998 [99]	AWs inflammation in swimmers	Elite swimmers and nonathletic control subjects	Comparison of baseline samples between swimmers (T: 800–3380 km/year) and control subjects	IS	>Eosinophils, >PMNs, >[EPO], and >[human neutrophil lipocalin] in swimmers in comparison to control subjects

Helenius et al. 2002 [100]	Retirement from swimming in relation to AWs inflammation	High performance swimmers	Comparison of baseline samples between active (T: ~1870 km/year) and inactive swimmers (3 months of inactivity)	IS	>eosinophils and >lymphocytes in active swimmers than inactive swimmers

Karjalainen et al. 2000 [101]	Inflammatory cells in skiers, mild asthmatics, and healthy control subjects^*∗*^	Elite healthy skiers and nonathletic control subjects	Comparison of baseline samples between skiers (T: 200–630 h/year) and control subjects	Endobronchial biopsy	>lymphocytes-T (43 times), >MØ (26 times), >eosinophils (2 times), and >PMNs (2 times) in skiers in comparison to control subjects

Martin et al. 2012 [102]	AWs inflammation and exposure to swimming pool in athletes^*∗*^	Endurance athletes	Comparison of baseline samples of pool based (5 h/wk) and non-pool-based (0.5 h/wk) athletes (T: ~15 h/wk)	EB and IS	PMNs and eosinophils in IS and eNO in EB were not different between groups

Sue-Chu et al. 1999 [103]	AWs inflammation in skiers	Cross-country skiers and nonathletic control subjects	Comparison of baseline samples during the competitive period, in autumn and winter, between skiers (T: 435 h/year) and control subjects	BALF	>total cells, >lymphocytes, and >mast cells in skiers in comparison to control subjects, with no differences in [TNF-*α*] and [MPO]

Sue-Chu et al. 2000 [104]	Budesonide and AWs inflammation in skiers^*∗*^	Elite cross-country skiers with asthmatic symptoms and budesonide or placebo supplementation	Comparison of baseline samples among skiers, after 20 weeks of supplementation with 800 *µ*g/d budesonide (T: ~427 h/year) or placebo (T: ~468 h/year)	BALF and endobronchial biopsy	Lymphocytes, MØ, eosinophils, PMNs, and mast cells were not different between groups

AWs: airways; BALF: bronchoalveolar lavage fluid; EB: exhaled breath; EBC: exhaled breath condensate; EPO: eosinophil peroxidase; H_2_O_2_: hydrogen peroxide; IS: induced sputum; 8-isoprostane PGF2*α*: 8-isoprostane prostaglandin F_2_ alpha; MØ: macrophages; MPO: myeloperoxidase; NO: nitric oxide; PMNs: polymorphonuclear neutrophils; T: training volume; TNF-*α*: tumor necrosis factor-alpha. In “*Aim,*” ^*∗*^the effect of exercise was not the primary aim of the study.

**(a):** 

Author, year	Aim	Sample characteristics	Exercise protocols	Samples obtained	Oxidative or inflammatory main results
Akil et al. 2015 [105]	Se administration affects lipid peroxidation in liver and lung tissues of rats subjected to acute swimming exercise^*∗*^	Sprague-Dawley adult male rats divide into general control, Se-administered, swimming control, and Se-administered swimming groups	Swimming was performed once for 30 minutes	Lung tissue	PE: ↑ MDA and ↑ GSH in swimming control versus general control

Al-Hashem 2012 [106]	VitE and VitC in protection of pulmonary damage induced by exercise in altitude^**∗**^	Wistar rats with 6 months of altitude adaptation	Forced swimming for 2.5 h in glass tank at 600 and 2270 MASL in accordance with altitude adaptation	Lung tissue	PE: ↑ [TBARs], ↓ SOD, and CAT activity at 600 MASL Supplementation with VitE and VitC reversed these results

Caillaud et al. 1999 [107]	Effect of acute exercise on lipid peroxidation in lung compared with locomotor muscles^*∗*^	Wistar rats exercised (E) and control rats (C)	Race on treadmill at 28 m/min and 15% grade (80–85 VO_2_max) until exhaustion (~66 min)	Lung tissue	PE: no changes of pulmonary activity of SOD, CAT, and [MDA] of E in comparison to C

Cathcart et al. 2013 [108]	Effects of exercise during different ambient temperatures and humidity on eNO, eCO, and pH	Thoroughbred racehorses	Exercised under saddle on an all-weather 1.6 km track at half-pace canter, full-pace canter, or gallop according to the current training regimen for each horse	EBC and EB	PE: only ↑ pH in EBC

Hatao et al. 2006 [109]	Acute exercise and antioxidant enzyme activation in aged rats^*∗*^	Young rats (YR) or aged rats (AR) exercised (E) or not exercised control (C)	Race on treadmill at 25 m/min for YRE and 18–20 m/min for ARE for 60 min	Lung tissue	PE: ↑ Mn-SOD activity in YRE and ARE in comparison to their control subjects; ↑ CuZn-SOD and CAT activity in YRE and ↓ reactive carbonyls derivative in ARE, in comparison to their control subjects

Huang et al. 2008 [110]	Supplementation with L-Arg on pulmonary inflammation and oxidative damage induced by exercise in aged rats^*∗*^	Sprague-Dawley rats exercised (E) or sedentary (S) with L-Arg (+L-Arg) or without control rats L-Arg (C)	Race on treadmill for groups E at ~70% VO_2_max until exhaustion (time for E+L-Arg and EC ~63 and ~51 min, resp.)	Lung tissue	PE: ↑ [XO], ↑ [MPO], and ↑ [MDA] in EC in comparison to SC; with no changes between EC and SC for [SOD], [CAT], [GSH-Px], [GR], and [GSH]

Kirschvink et al. 2002 [13]	Oxidative state, pulmonary function, and airway inflammation in healthy horses and with arcades^*∗*^	Trained healthy horses, affected by arcades or clinical remission	Race on treadmill with 2 min to 8, 9, and 10 m/s and 4% inclination, stages interrupted by 2 jogs of 8 min to 3.5 m/s (10 min of warming up and 10 min of recovery)	BALF	PE: ↑ [UA] in healthy horses

Lin et al. 2005 [111]	Oxidative stress and antioxidant defenses in animals supplemented or not with L-Arg^*∗*^	Sprague-Dawley rats grouped as exercised (E) or sedentary (S) with L-Arg (+L-Arg) or control rats without L-Arg (C)	Race on treadmill for E groups at 20 m/min for 15 min and 25 m/min for 30 min; then they run at 30 m/min and 10% of inclination (70–75% VO_2_max) until exhaustion (EC ~81 min and E+L-Arg ~87 min)	Lung tissue	PE: ↑ activity XO and MPO in EC in comparison to SC; ↑ [UA], ↑ [NO], and ↑ [MDA] in EC in comparison to SC; ↑ activity SOD and GR in EC in comparison to SC

Mills et al. 1996 [112]	eNO and VNO during acute exercise	Healthy horses	Maximal incremental race until 9 m/s	EB	DE: positive correlation of eNO and VNO with the race intensity

Radák et al. 1998 [113]	Acute anaerobic exercise and oxidative modification of pulmonary proteins	Exercised Wistar rats (E) and sedentary control rats (C)	Two races on treadmills at 30 m/min for 5 min; after 5 min of recovery, a 3rd race to exhaustion was performed	Lung tissue	PE: >pulmonary carbonyls and [glutamine synthetase] in E versus C

Reddy et al. 1998 [114]	Pulmonary oxidative damage by acute strenuous exercise in rats deficient in Se and VitE	Female Wistar albino rats deficient in Se and VitE and control rats	Intense swimming to exhaustion	Lung tissue	PE: >[SOD] and <[GSH-Px] and <[GST] in rats deficient in VitE and in comparison to control rats

Prigol et al. 2009 [115]	Supplementation with (PhSe)_2_ and pulmonary oxidative damage caused by the exercise	Adult Swiss albino mice supplemented with (PhSe)_2_ and not supplemented control mice	Swimming exercise (20 min) for both groups after 7 d of supplementation	Lung tissue	PE: ↑ [MDA] and ↑ of CAT activity in mice not supplemented with (PhSe)_2_

Terblanche 1999 [116]	Exhaustive swimming and CAT activity in the lungs of male and female rats^*∗*^	Sprague-Dawley rats	1 h swimming	Lung tissue	PE: ↑ CAT activity in males and females

BALF: bronchoalveolar lavage fluid; CAT: catalase; (PhSe)_2_: diphenyl diselenide; GR: glutathione reductase; GSH: glutathione reduced; GSH-Px: glutathione peroxidase; GST: glutathione S-transferase; L-Arg: L-arginine; MASL: meters above sea level; MDA: malondialdehyde; MPO: myeloperoxidase; NO: nitric oxide; Se: selenium; SOD: superoxide dismutase; CuZn-SOD: copper-zinc-superoxide dismutase; Mn-SOD: manganese-superoxide dismutase; TBARs: thiobarbituric acid reactive substances; UA: uric acid; VNO: volume of nitric oxide; XO: xanthine oxidase; VitE: vitamin E; VitC: vitamin C. In “*Oxidative or inflammatory main results,*” DE: during exercise and PE: postexercise. In “*Aim,*” ^*∗*^the effect of exercise was not the primary aim of study.

**(b):** 

Author, year	Aim	Sample characteristics	Exercise protocols	Samples obtained	Oxidative or inflammatory main results
Altan et al. 2009 [117]	SOD activity and [TBARs] postadaptation by training in altitude^**∗**^	Wistar albino rats divided into trained in hypobaria (THb) and normobaria (TNb) and nontrained in hypobaria (Hb) and normobaria (Nb)	Comparison of baseline samples between groups trained with swimming (T: 5 at 30 min/day/for 4 days/week for 9 weeks) or nontrained and exposed or not to simulated altitude of 3000 MASL (E: 120 min/day for 4 days/week for 9 weeks)	Lung tissue	PT: >SOD activity in TNb in comparison to Nb; no differences in [TBARS] for the same groups

Asami et al. 1998 [118]	DNA oxidative damage by chronic exercise	Sprague-Dawley rats with spontaneous (S), forced (F) exercise and sedentary control rats (C)	Comparison of baseline samples among rats with spontaneous exercise (wheel), trained on treadmill (T: 30–90 min/day for 25 days), and control rats	Lung tissue	PT: >[8-OH-dG] in F in comparison to S; the DNA oxidative damage was related to the exercise intensity

Aydin et al. 2009 [119]	Long period of dietary restriction and stress produced by high intensity swimming^**∗**^	Sprague-Dawley rats with restricted diet (RD) or ad libitum (AL), grouped in trained (+T), exercised (+E), and sedentary control rats (C)	Comparison of baseline samples of RD and AL in +T (T: 8 weeks of swimming with 2% BW as extra load during ~50–80 min), PE in +E (E: swimming until exhaustion), and baseline C	Lung tissue	PT: <GSH activity and >GSH-Px of AL+T compared to ALC; <LPO, >GSH, and GSH-Px in AL+E that AL+T PE: ↑ [MDA], ↓ [GSH], ↓ GR activity, and ↑ GSH-Px of AL+E compared to ALC (acute effects)

Chimenti et al. 2007 [120]	Epithelial remodeling, inflammatory cells, and apoptosis in the AWs after chronic exercise	Trained Swiss mice (T) and sedentary control mice (C)	Comparison of baseline samples among trained mice (T: 5 d/week for 6 wk at moderate to high intensity)	Lung tissue	PT: >apoptosis, >proliferation, >loss of hair cells, and infiltration of leukocytes in the AWs in T versus C

da Cunha et al. 2013 [121]	Chronic exercise on oxidative stress and NF-к*β*/p65 pulmonary immunocontent of rats with lung injury	Trained Wistar rats (T) and nontrained control rats (C)	Comparison of baseline samples among rats trained on treadmill (T: 20 min at 60% VO_2_max during 24 days in 3 months)	BALF and lung tissue	PT: >pulmonary catalase activity in T versus C; there are no changes in [TBARs], carbonyls, dichlorofluorescein, [NO_2_^−^], and NF-к*β*/p65 in the lung

Gündüz et al. 2004 [122]	Oxidant and antioxidant systems in rats organs after a year of training^**∗**^	Wistar albino rats grouped in young control rats (YC), aged control rats (AC), and aged rats-training (AT)	Comparison of baseline samples between AT in swimming (T: 1 h/day for 5 days/week for 1 year) with YC and AC	Lung tissue	PT: >SOD activity and >GSH-Px in AT in comparison to AC; no difference of [TBARs] between the same groups

Lee et al. 2013 [123]	Administration of a ginseng intestinal metabolite (IH901) and exercise-induced oxidative stress in trained rat^**∗**^	Sprague-Dawley rats divided into resting control (RC), training control (EC), resting with IH901 consumption, or exercise with IH901 consumption groups	Training was carried out during 8 weeks on a treadmill; two weeks with 0% inclination and 25 cm/sec; then 2 weeks with 10% and 30 cm/sec; then 4 weeks with 15% and 35 cm/sec	Lung tissue	PT: ↑ TBARs and ↑ protein carbonyls in EC versus RC

Menegali et al. 2009 [124]	Therapeutic effects of physical exercise on histological and oxidative stress markers in animals exposed to cigarette smoke^**∗**^	Old C57BL-6 mice divided into control (C), training (T), cigarette smoke (CS), and cigarette smoke plus training (CS+E) groups	Training groups swam for 10 min/day during one habituation week; then they performed a swimming program 5 days/week for 8 weeks	Lung tissue	PT: ↑ SOD and ↑ CAT activity in E versus C

Olivo et al. 2014 [125]	Moderate aerobic exercise training prior to *Streptococcus pneumoniae* infection influences pulmonary inflammatory responses^**∗**^	BALB/c mice divided into sedentary untreated (SU), sedentary infected (SI), aerobic trained untreated (ATU), and aerobic trained infected groups (ATI)	Comparison between SU and ATU during 4 weeks after an individual maximal exercise capacity test was performed (0.1 km/h every 2.5 min, 25% inclination); training was for 60 min/day, 5 days/wk for 4 wk at 50% of the maximal speed	BALF and lung tissue	PT: ↑ CuZn-SOD and ↑ Mn-SOD expression in lung parenchyma of ATU versus SU after an individual maximal exercise capacity test

Reis Gonçalves et al. 2012 [15]	Chronic aerobic exercise on pulmonary inflammation, cytokine, and antioxidant enzymes in animal model of acute pulmonary damage^**∗**^	Trained BALB/c mice	Comparison of samples before and after a low intensity training on treadmill (T: 50% of MS for 60 min/d, 3 d/week for 5 weeks)	BALF, EB, and lung tissue	PT: with no changes in leukocytes, [IL-6], [IL-10], nor [TNF-*α*] in BALF; with no changes in [NO] in EB; ↑ expression of IL-6 and Mn-SOD in the lung, but no changes of activity of GSH-Px and GR in the lung

Toledo et al. 2012 [126]	Regular physical exercise in an experimental mouse model exposed to cigarette smoke^**∗**^	C57BL/6 mice divided into control mice (C), trained (T), exposed to cigarette smoke (Sk), and Sk plus T (Sk+T)	Comparison of baseline samples in T at moderate intensity on treadmill (T: 50% MS for 60 min/d, 5 d/week for 24 weeks)	BALF and lung tissue	PT: <[ROS] in BALF of En compared to C; >GSH-Px activity, but not of Mn-SOD nor CuZn-SOD in lungs of T compared to C; with no changes in the expression of IL-1ra, TNF-*α*, and IL-10 between T and C

Yang 2011 [127]	Chronic exercise and expression of cytokines related to inflammation in the lung tissue	Old male Sprague-Dawley rats, group with trained rats (T) and sedentary control rats (C)	Comparison of baseline samples between rats trained on treadmill (T: 25 m/min for 120 min/day for 1 week) and control rats	Lung tissue	>expression of mRNA for TNF-*α* and IL-4 and <expression of mRNA for IFN-*γ* of group T versus C

BALF: bronchoalveolar lavage fluid; BW: body weight; DEP: diesel exhaust particles; DNA: deoxyribonucleic acid; EB: exhaled breath; 8-OH-dG: 8-hydroxydeoxyguanosine; GR: glutathione reductase; GSH: glutathione reduced; GSH-Px: glutathione peroxidase; IFN-*γ*: interferon gamma; IL-1ra, IL-4, IL-6, or IL-10: interleukin-1ra, interleukin-4, interleukin-6, or interleukin-10; LPO: lipid peroxidation; MDA: malondialdehyde; MS: maximal speed; mRNA: messenger RNA; MS: maximal speed; NF-к*β*/p65: factor nuclear kappa-*β*/p65; NO: nitric oxide; NO_2_^−^: nitrite; ROS: reactive oxygen species; SOD: superoxide dismutase; CuZn-SOD: copper-zinc-superoxide dismutase; Mn-SOD: manganese-superoxide dismutase; TBARs:  thiobarbituric acid reactive substances; TNF-*α*: tumor necrosis factor-alpha. In “*Oxidative or inflammatory main results,*” PE: postexercise and PT: posttraining. In “*Aim,*” ^*∗*^the effect of exercise was not the primary object.
